# Comparison between a Conductometric Biosensor and ELISA in the Evaluation of Johne's Disease

**DOI:** 10.3390/s141019128

**Published:** 2014-10-15

**Authors:** Chika Okafor, Daniel Grooms, Evangelyn Alocilja, Steven Bolin

**Affiliations:** 1 Large Animal Clinical Sciences, Michigan State University, East Lansing, MI 48824, USA; E-Mail: groomsd@cvm.msu.edu; 2 Biosystems and Agricultural Engineering, Michigan State University, East Lansing, MI 48824, USA; E-Mail: alocilja@msu.edu; 3 Diagnostic Center for Population and Animal Health, Michigan State University, East Lansing, MI 48824, USA; E-Mail: bolins@dcpah.msu.edu

**Keywords:** biosensor, conductometric, diagnosis, immunosensor, Johne's disease, paratuberculosis

## Abstract

Johne's disease (JD), caused by *Mycobacterium avium* subspecies *paratuberculosis* (MAP), is an important gastrointestinal disease of cattle worldwide because of the economic losses encountered in JD-affected herds. These losses include reduction in milk yield in cows, premature culling and reduced carcass weight of culled diseased animals. In the U.S. dairy industry, economic losses from reduced productivity associated with JD are estimated to cost between $200 and $250 million annually. The development of non-laboratory-based assays would support more frequent testing of animals for JD and could improve its control. Conductometric biosensors combine immunomigration technology with electronic signal detection and have been adapted for the detection of IgG antibody against MAP. In the present study, a capture membrane with limited variability in the immunomigration channel and an optimal concentration of the secondary anti-bovine antibody used in a previously developed conductometric biosensor were compared with a commercially available antibody detection ELISA in their evaluation of JD, using samples of serum from cattle whose JD status where unknown. There was a moderate strength of agreement (kappa = 0.41) between the two assays. Findings from this preliminary study support the continued development of conductometric biosensors for use in the diagnosis of JD.

## 1. Introduction

Johne's disease (JD) is a chronic gastrointestinal disease of animals, especially domestic ruminants. It is caused by *Mycobacterium avium* subsp. *paratuberculosis* (MAP), and young animals are most susceptible to MAP infection. JD animals shed viable MAP in their milk and feces. The disease causes a significant economic impact on the global cattle dairy industry [1], mainly from the effects of reduced milk production [2]. In the US dairy industry, economic losses from reduced productivity associated with JD have been estimated to be between $200 and $250 million annually [3]. JD also raises public health concerns, because MAP infections have been reported in some Crohn's disease patients [4,5]. Some evidence exists that MAP may be associated with Crohn's disease in humans; however, causality criteria have not been met, and currently, MAP is not recognized as a zoonotic pathogen [6]. With the economic losses from JD and the possibility that MAP may be a zoonotic pathogen, early detection of JD-affected animals at points-of-concentration, such as sale barns, could help in reducing disease spread. The commonly used tests for JD diagnosis—bacterial culture, polymerase chain reaction (PCR) assay and enzyme-linked immunosorbent assay (ELISA)—are not suitable for cow-side diagnosis [7]. Therefore, developing rapid cow-side diagnostic assays, which can be readily deployed in the field, could aid in furthering the control efforts of JD.

Biosensors are among the new pathogen detection or disease diagnostic assays in biomedical sciences that have potential advantages, including, rapid detection, portability and adaptability for patient-side use [8,9]. Among the different types of biosensors [7,9,10], a conductometric biosensor is an analytical device that contains a transducer, which interprets specific biological recognition reactions (*i.e.*, antigen-antibody binding) as electrical conductance. A transducer, such as polyaniline, is placed close to or integrated with the biological element (*i.e.*, antibody). Polyaniline, a conductive polymer, relays any antigen-antibody binding as a measured electrical quantity on a detector instrument. Conductometric or other lateral flow biosensors have been used to detect microbial agents, such as *E. coli* O157:H7 [11,12], *Bacillus cereus* [13], bovine viral diarrhea virus (BVDV) [14], antibodies against MAP [7] or MAP organisms [15]. The developed biosensor for MAP immunoglobulin G (IgG) detection possesses some desirable attributes, such as relative rapidity in detection and on-site adaptability, which could make it a useful assay for JD control. However, optimization of this biosensor is needed to improve its precision and accuracy.

The objectives of this study were to: (1) optimize the anti-bovine antibody concentrations of a previously developed conductometric biosensor for detecting MAP IgG using a capture membrane with a uniform immunomigration channel; and (2) compare JD results obtained using the biosensor and those obtained using a commercially available ELISA. By comparing the improved biosensor with a similar immunodiagnostic assay, the ELISA, the usefulness of the former as a diagnostic assay for JD can be assessed. The outcome of this study would help evaluate possible modifications that can improve the usefulness of the conductometric biosensor in JD diagnosis and control programs.

## 2. Experimental Section

The biosensor used consists of an immunosensing component and a signal detector system. The immunosensing component comprises four individual membranes: sample application, conjugate, capture and absorption membranes (Hi-Flow Plus Assembly Kit, Millipore, Bedford MA, USA). The suitability of the immunosensor membranes, the silver electrodes and assembling of the biosensor assay have been reported previously [7]. Hence, major differences with that previous work are reported in the present study.

### 2.1. Capture Membrane Preparation

**Figure 1.**
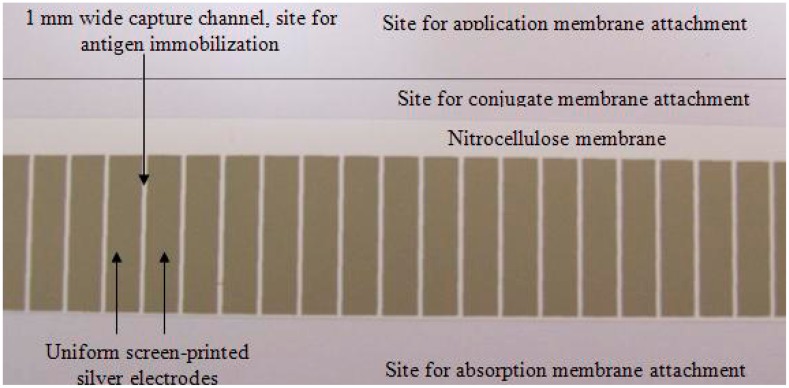
Screen-printed silver electrode films on the capture membrane before immunosensor assembly and cutting.

In the present study, silver electrodes were screen-printed on the membrane earlier in the preparation process to yield several 1 mm-wide capture channels (Figure 1). The rest of the capture membrane preparation was performed as was described in the previous study.

### 2.2. Optimization of Anti-Bovine Antibody Concentrations in Polyaniline Conjugate

AquaPass polyaniline (Pani) (Mitsubishi Rayon Co, Tokyo, Japan) was diluted to 0.001% with 0.1 M phosphate buffer saline (PBS). Purified mouse monoclonal anti-bovine IgG (clone BG-18) (Sigma-Aldrich, St Louis, MO, USA) was added to 0.001% Pani solution to produce 3 final concentrations (w/v) of monoclonal anti-bovine IgG (AB/IgG*): 0.046 mg/mL, 0.0115 mg/mL and 0.0046 mg/mL. A 4-mL aliquot of each concentration of AB/IgG* in Pani solution was incubated at 27 °C for 1.0 h to form Pani-AB/IgG* conjugate. Afterwards, a blocking solution consisting of 0.5 mL of 0.1 M Tris buffer containing 0.1% casein (pH 9.0) was added to the each Pani-AB/IgG* conjugate solution and incubated at 27 °C for 0.5 h. To achieve immobilization, the conjugate membrane was immersed in the Pani-AB/IgG* conjugate and blocking solution until the membrane was saturated. Then, the membrane was air-dried at 20 °C under a clean biosafety cabinet for 0.75 h.

### 2.3. Biosensor Assembly and Mechanism of Detection

The membranes used for sample application, conjugate reservoir, antigen capture and liquid absorption were assembled into an immunosensor, as was described in the earlier study. Three separate immunosensors representing each of the various AB/IgG* concentrations were individually assembled. Each assembled immunosensor was cut into 5 mm-wide disposable strips. A silver-microtip conductive pen (MG Chemicals, Surrey, BC, Canada) was used to hand-print a connection between each silver electrode flanking the 1-mm strip of antigen capture membrane and a copper wafer. Each end of the copper wafer was connected to an ohmmeter (Model: 2880A BK Precision multimeter, Worchester, MA, USA), which was the detector element. The sample to be tested (100 μL) was applied to the application membrane. The sample was drawn into the 1-mm channel of the immunosensor strip by capillary action, and the schematic of the assay detection was presented in the previous study. Descriptively, as the sample passed over the conjugate membrane, serum IgG bound with the Pani-AB/IgG* conjugate, forming Pani-AB/IgG*-IgG complex. The complex was pulled onto the capture membrane, where immobilized antigen (MAPPD) captured antibody directed against MAP. The remaining non-MAP-specific IgG flowed to the absorption membrane. As more and more MAP-specific antibodies were captured, the polyaniline in the Pani-AB/IgG*-IgG complex formed a bridge between the silver electrodes that flank the antigen capture membrane. The polyaniline caused an electrical conductance through the electrodes, which was recorded as a reduced resistance for an electrical current.

### 2.4. Samples

Initially, each biosensor was tested with a negative control (0.1 M PBS) and with 6 samples of bovine serum that were tested in the previous study: 3 sera from JD-positive cows (clinical JD cows that were housed at the Michigan State University Veterinary Research Farm, East Lansing, MI, USA) and 3 sera from JD-negative cows (persistent JD-negative cows that triple-tested negative for JD at the Michigan State University Dairy Teaching and Research Center, East Lansing, MI, USA). The JD status of the samples was determined previously using a commercially available MAP antibody ELISA (PARACHEK, Prionics, Schlieren-Zurich, Switzerland). The ELISA interpretation was based on the optical density (OD) values following the recommendations of the manufacturer. Using the aforementioned 7 samples, the optimal concentration of anti-bovine antibody conjugated with polyaniline needed for detection of antibody against MAP was determined. After the optimization, 17 untested bovine serum samples, a positive control and 2 negative controls, were used to test the diagnostic potential of the biosensor with respect to JD. The 17 samples were frozen serum specimen previously collected from a Michigan dairy farm infected with JD animals. An aliquot of each collected serum was evaluated for JD separately on the biosensor and on a commercially available MAP antibody ELISA, and the obtained results were compared for agreement.

### 2.5. Signal Measurement

After each sample application, the resistance value (kiloohms) was recorded at 2 min, based on the results of the previous study. Three replications were performed on each sample. The samples used for the optimization step were tested on three separate biosensors with varying AB/IgG* concentrations. After the optimization, the unknown serum samples were tested on the biosensor with 0.0115 mg/mL of AB/IgG* in Pani.

### 2.6. Statistical Analyses

For the optimization step, each biosensor's intra-assay coefficient of variation (%CV) was calculated to evaluate if the uniformly screen-printed electrodes affected the precision of the biosensor assay. A 2-way ANOVA was used to analyze if the mean resistance values among the sample groups were significantly different, adjusting for the effects of different ELISA OD values and different AB/IgG* concentrations; the Holm–Sidak test, a multiple comparison procedure, was used to isolate which group(s) differed from the others. These statistical analyses were performed with SigmaStat 3.1 software (Systat Software Inc., San Jose, CA, USA).

The %CV evaluation was also performed on the blinded serum samples. The biosensor's cut-off value was determined using the mean ± 2SD [16]. The strength of agreement between the assays was analyzed using Cohen's kappa analysis on GraphPad software (GraphPad Software, Inc., La Jolla, CA, USA).

## 3. Results and Discussion

### 3.1. Results

#### 3.1.1. Optimization Step

The results of the AB/IgG* optimization assays are shown in Table 1. For biosensors made from 0.046 mg/mL, 0.0115 mg/mL and 0.0046 mg/mL AB/IgG* concentrations, the %CV was 4.90%, 3.88%, and 7.62% respectively. The conductometric biosensor evaluation of the samples, for each AB/IgG* concentration, showed numerically lower mean resistance values among the JD-positive samples than those observed in the JD-negative samples and the negative control.

**Figure 2.**
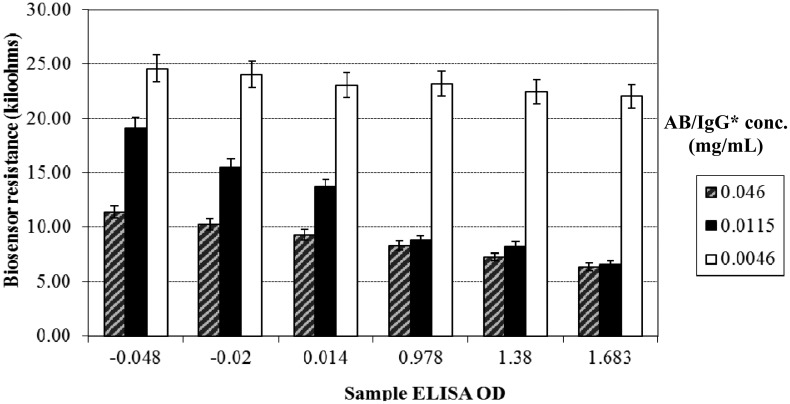
Mean biosensor resistance values of six serum samples at different AB/IgG* concentrations (mg/mL). ELISA OD <0.1 = JD negative, ≥0.1 = JD positive.

The mean resistance values among the sample groups were significantly different (*p* < 0.001). The observed resistance values were significantly affected by the ELISA OD values and the AB/IgG* concentrations (*p* < 0.001), both individually and by their two-way interactions. The Holm–Sidak test (Figure 2) showed that for a 0.0115 mg/mL AB/IgG* concentration, the mean resistance values of each of the JD-positive ELISA OD values (16.83, 13.80 and 9.76) was significantly different (*p* < 0.05) from each of the JD-negative ELISA OD values (0.14, −0.20 and −0.48). However, there was no significant difference between the two groups for the 0.046 mg/mL and 0.0046 mg/mL AB/IgG* concentrations (*p* > 0.05).

#### 3.1.2. Evaluation of Unknown Samples

With the mean ±2SD method, a cut-off value of 12.03 kiloohms (KΩ) (<12.03 = JD positive, ≥12.03 = JD negative) was generated. Of the 17 tested serum samples, there were five positive and seven negative concordant results between the biosensor and ELISA in the diagnosis of JD; however, two negative and three positive biosensor results were discordant with the ELISA's (Table 2). Cohen's kappa value was 0.41 at the 95% confidence interval, and the intra-assay %CV of the biosensor was 5.91%.

### 3.2. Discussion

Although our previously developed biosensor was capable of detecting antibody against MAP, the intra-assay variability (%CV = 14.48%) was high. To address variability in the preparation of the biosensor electrodes, uniform screen-printed electrodes replaced the hand-printed electrodes used in the previous study. The screen-printed electrodes yielded a consistent 1 mm-wide capture channel on the capture membrane of the conductometric biosensor. The intra-assay %CV of each of the optimized biosensors was less than 8%. A reasonable target for %CV in routine diagnostic testing is 10%–15%; however, a %CV lower than 10% is a good indication of precision, which is a desired attribute in assay validation [17].

To further optimize the biosensor, the effect of different concentrations of AB/IgG* on the biosensor performance was evaluated. The difference in resistance between JD-positive and JD-negative samples was significant (*p* < 0.05) when 0.0115 mg/mL of AB/IgG* was used, but not when 0.046 mg/mL and 0.0046 mg/mL AB/IgG* were used. At the lowest AB/IgG* concentration (0.0046 mg/mL), the relatively higher resistance in all samples could be explained by too few molecules of antibody conjugate bound to antigen on the capture membrane to effectively complete an electrical circuit across the silver electrodes. Hence, the few immobilized MAP-specific Pani-AB/IgG*-MAP antigen complexes would result in relatively low electrical conductance, leading to a relatively high resistance value. The relative higher resistance values with lower AB/IgG* concentrations were seen in a similar study, where a conductometric biosensor was developed to detect BVDV [14].

For all of the sample groups, the higher AB/IgG* concentration biosensor (0.046 mg/mL) yielded lower resistance values than the other AB/IgG* concentration biosensors. The excess AB/IgG* molecules after Pani-AB/IgG* conjugation could be responsible for lower resistance values at higher AB/IgG* concentrations. Upon sample application, the excess un-conjugated AB/IgG* molecules could get attached to serum IgG to form AB/IgG*-IgG molecules. In the capture membrane, the AB/IgG*-IgG molecules could crowd the capture channel, such that the crowded molecules could provide a platform for easier bridging of the electrodes, inducing higher conductance (lower resistance). This over-crowding effect may be responsible for the low resistance observed at the 0.046 mg/mL AB/IgG* concentration. Given the parameters used in this study, the 0.0115 mg/mL AB/IgG* concentration was optimum for the biosensor's detection of MAP IgG.

The relative resistance values recorded in this study for the 0.0115 mg/mL AB/IgG* concentration was lower in comparison to the previous study, and the reason for this change in resistance values is not yet understood. The few areas of change in methodology could be responsible. One possible reason could be that the conductive properties of the silver paste used for electrode printing in this study differed from that of the silver ink used in the previous study. Another reason could be the effect of the roller, in the programmable shear, pressing on the immunosensor membranes during membrane cutting. Pressing on the capture membrane after MAPPD immobilization could push the antigen into the nitrocellulose membrane or distort the antigen's orientation, thereby affecting specific antigen-antibody binding. Cutting of the assembled immunosensor membranes without tampering with the membrane surfaces might address the resistance change.

When the biosensor was used to examine unknown serum samples and compared with a commercially available antibody ELISA, a Cohen's kappa value of 0.41 signified a moderate strength of agreement between the two assays. The biosensor had a good level of precision (intra-assay coefficient of variation 5.91%). However, there were increased variations in the results obtained from Samples 9, 10 and 12 (Table 2), such that the results could be interpreted as negative or positive for JD. In each of the three affected samples, a lone reading from the triplicate results caused those levels of variations. For each sample, two readings were suggestive of a negative interpretation, while one reading was off. The supposed outliers in these three samples were not retested to avoid the possibility of a biased outcome. ELISA was chosen as the assay for comparison, because it is most commonly used in the cattle industry today [18]. However, ELISA has a 30% ± 5% sensitivity and a 99.5% ± 1% specificity in reference to necropsy results for JD [19]. The relatively low sensitivity of ELISA could make it difficult for a generalized evaluation of the biosensor, based on the obtained kappa value in this study. Either fecal culture, the most sensitive (60% ± 5%) and specific (99.9% ± 0.1%) of the JD tests when necropsy was used as a gold standard [19], or necropsy should be a better test to evaluate the diagnostic ability of the biosensor. Nevertheless, a broader generalization of the results obtained in this study is limited by the number of samples tested and by not absorbing the sera with *M. phlei* prior to testing with biosensors. Although this latter step would help improve the specificity of the assay, it could hinder the adaptability of the biosensor assay as a cow-side test for JD diagnosis and may not be too important if the desired use of this assay is to improve the sensitivity of JD diagnosis at a fairly short time interval. The adaptability of the biosensor as a cow-side screening assay at points-of-concentration where timely knowledge of JD status is important is a significant advantage of the biosensor over ELISA. Although, the biosensor as described in this study is quite tedious, the process can be commercialized into ready-to-use immunomigration strips when the potential benefits of the biosensors are justified. Further characterization of the biosensor using a well-characterized increased number of serum samples from true JD-positive and true JD-negative animals is necessary. Areas for further research are optimizations of MAP antigen types, MAP antigen concentrations, sample type (milk, whole blood), sample processing and polyaniline concentrations.

## 4. Conclusions

In this study, a conductometric biosensor for MAP-specific antibodies was further optimized and shown to have moderate agreement with commercially available antibody detection ELISA's. The biosensor assay is a promising tool that could be used in the control of JD in cattle. Additional optimization of the biosensor assay could further improve its precision and accuracy, making it a desirable assay for enhancing JD control.

## Figures and Tables

**Table 1.** Results of conductometric biosensor analysis of bovine serum samples at different concentrations of anti-bovine IgG conjugated with polyaniline.

**Corrected ELISA OD Value**s	Conductometric Biosensor Resistance (KΩ) at 2 min for Varying AB/IgG* **Concentrations in 0.001% AquaPass**		

	**0.046 mg/mL Mean** ± **SD**	**0.0115 mg/mL Mean** ± **SD**	**0.0046 mg/mL Mean** ± **SD**
1.683 **	6.35 ± 0.12 ^a^	6.56 ± 0.31 ^a^	22.01 ± 1.70 ^a^
1.380 **	7.24 ± 0.68 ^a^	8.23 ± 0.34 ^a^	22.46 ± 0.90 ^a^
0.978 **	8.30 ± 0.46 ^a^	8.80 ± 1.36 ^a^	23.19 ± 2.45 ^a^
0.014 *	9.30 ± 0.33 ^ab^	13.70 ± 0.27 ^b^	23.04 ± 2.60 ^a^
−0.020 *	10.25 ± 0.45 ^ab^	15.52 ± 0.28 ^b^	24.04 ± 2.43 ^a^
−0.48 *	11.39 ± 0.55 ^b^	19.13 ± 0.23 ^c^	24.59 ± 0.54 ^a^
(−) control PBS	14.94 ± 1.06 ^c^	20.73 ± 1.80 ^c^	24.28 ± 0.34 ^a^

** Johne's disease (JD)-positive, corrected ELISA OD >0.1;

* JD-negative, corrected ELISA OD value ≤0.1; SD, standard deviation; different superscripts ^a,b,c^ within the columns indicate significant differences between the mean resistance of the samples (*p* < 0.05).

**Table 2.** Comparison between a conductometric biosensor and ELISA in the evaluation of Johne's disease.

**Sample ID**	**Mean** ± **SD Biosensor Resistance (KΩ)**	**Biosensor Interpretation**	**ELISA OD**	**ELISA Interpretation**
1	9.59 ± 1.34	Positive	0.00	Negative
2	10.17 ± 0.29	Positive	0.17	Positive
3	10.37 ± 0.30	Positive	0.98	Positive
4	10.70 ± 0.07	Positive	0.46	Positive
5	10.74 ± 0.05	Positive	0.50	Positive
6	10.76 ± 0.06	Positive	0.54	Positive
7	10.98 ± 0.07	Positive	0.00	Negative
8	11.66 ± 0.61	Positive	0.00	Negative
9	12.14 ± 1.10	Negative	0.00	Negative
10	12.27 ± 1.64	Negative	0.93	Positive
11	12.40 ± 0.33	Negative	0.00	Negative
12	12.84 ± 1.56	Negative	0.00	Negative
13	13.30 ± 0.31	Negative	0.00	Negative
14	13.38 ± 0.24	Negative	0.00	Negative
15	13.64 ± 0.55	Negative	0.00	Negative
16	14.80 ± 1.02	Negative	0.61	Positive
17	15.70 ± 3.01	Negative	0.00	Negative
(+) control	5.61 ± 0.30	Positive	1.68	Positive
(−) control	12.77 ± 0.37	Negative	−0.02	Negative
0.1 M PBS (−) control	21.81 ± 1.46	Negative	N/A	N/A

SD, standard deviation; OD, optical density; PBS, phosphate buffered saline.
